# Therapeutic Drug Monitoring in Patients with Systemic Lupus Erythematosus: Utility and Gaps

**DOI:** 10.3390/jcm13020451

**Published:** 2024-01-13

**Authors:** Kar Mun Chong, He Jiang, Elaine Ah Gi Lo, Wei-Zhen Hong, Emmett Tsz-Yeung Wong, Gek Cher Chan, Jiacai Cho

**Affiliations:** 1 Division of Rheumatology, Department of Medicine, National University Hospital, Singapore 119074, Singapore; karmun.chong@mohh.com.sg; 2 Department of Pharmacy, National University Hospital, Singapore 119074, Singapore; he_jiang@nuhs.edu.sg (H.J.); elaine_lo@nuhs.edu.sg (E.A.G.L.); 3 Division of Nephrology, Department of Medicine, National University Hospital, Singapore 119074, Singapore; wei_zhen_hong@nuhs.edu.sg (W.-Z.H.); emmett_ty_wong@nuhs.edu.sg (E.T.-Y.W.); gek_cher_chan@nuhs.edu.sg (G.C.C.); 4 Department of Medicine, Yong Loo Lin School of Medicine, National University of Singapore, Singapore 117597, Singapore; 5 National University Centre for Organ Transplantation, National University Hospital, Singapore 119074, Singapore

**Keywords:** systemic lupus erythematosus, therapeutic drug monitoring, pharmacogenomics, hydroxychloroquine, mycophenolate, cyclosporine, tacrolimus, azathioprine

## Abstract

Despite advances in the treatment of patients with systemic lupus erythematous (SLE), outcomes have remained suboptimal. Persistent disease activity, patient comorbidities and drug toxicities contribute to the accrual of progressive irreversible damage and high rates of morbidity and mortality. Currently, similar drug doses and regimens are promulgated in the treatment guidelines for all SLE patients, despite the vast differences in patient and environmental factors that affect the drugs’ metabolism and blood concentrations. This causes a disconnect between drug dosing and drug blood concentrations, which can then result in unpredictability in drug toxicities and therapeutic effects. In this review, we discuss commonly used oral immunosuppressive medications in SLE, their pharmacogenomics, and factors affecting their metabolism and blood concentrations. Further, we highlight the role of therapeutic drug monitoring in SLE, which is the first accessible step to individualising therapy.

## 1. Introduction

Systemic lupus erythematosus (SLE) is a highly heterogenous disease with diverse clinical manifestations. It predominantly affects younger females with a median age of 30 years at diagnosis [1], but paediatric-onset and late-onset disease are also well established. With a global prevalence, there are higher rates of lupus nephritis in Asian patients [2], and overall worse outcomes in African American and Hispanic patients [3]. Lifelong therapy is required for the majority of patients as remission and durable remission are rare [4]. In comparison to the large spectrum of diseases caused by SLE, the options for treatment are disproportionately limited. This presents a need to individualize therapy in the everyday clinic to reduce toxicities and maximise efficacy. 

The drug levels of immunosuppressive therapies can be influenced by patient factors such as body weight, cytochrome P450 gene polymorphisms, renal and liver impairment, or environmental factors such as smoking and dietary polyphenols. In addition, SLE disease activity may also alter the pharmacokinetics of these drugs. For example, hypoalbuminemia from lupus nephritis or protein-losing enteropathy increase the unbound fraction of mycophenolic acid in the serum. The therapeutic monitoring of drug levels bypasses these limitations and allows clinicians to assess the actual drug concentrations independent of the prescribed doses. 

Therapeutic drug monitoring (TDM) involves the use of drug levels to guide dosing and is based on the premise that (1) there is a correlation between drug levels and therapeutic outcomes such as efficacy and safety and that (2) there is inter-individual variability in drug metabolism (pharmacokinetics) and the magnitude and duration of the response to therapy (pharmacodynamics). In this review, we aim to outline the factors that are known to alter the drug levels of hydroxychloroquine, azathioprine, mycophenolate and calcineurin inhibitors, the utility of monitoring the drug levels of these medications, as well as the gaps in current knowledge. 

## 2. Methods

We searched for English language articles in the electronic database PubMed using the Medical Subject Heading (MeSH) terms “systemic lupus erythematosus” and “therapeutic drug monitoring”, with no date restrictions. The content was further supplemented by important references such as UptoDate and StatPearls.

## 3. Hydroxychloroquine (HCQ)

HCQ is a key anchor drug in SLE: it improves overall survival [5], reduces the risk of all flares [6] and renal flares [7], improves the response to induction therapies in lupus nephritis [5], retards irreversible organ damage [8], especially cardiovascular damage, and reduces thrombosis in patients with associated antiphospholipid syndrome [9]. International guidelines from the European Alliance of Associations for Rheumatology (EULAR), the American College of Rheumatology (ACR) and the Kidney Disease Improving Global Outcomes (KDIGO) universally recommend the use of HCQ in all patients with SLE unless there are contraindications or side effects [10,11].

Yet, the continued use of HCQ and its dosing are not without controversies. In 2016, the American Academy of Ophthalmology (AAO) changed the recommended dosing of HCQ from ≤6.5 mg/kg/day (ideal body weight) to ≤5 mg/kg/day (actual body weight) [12]. This change was mainly driven by a retrospective review of 2361 patients who were on HCQ that reported a retinal toxicity of <1% after 5 years, <2% after 10 years, and almost 20% after 20 years [13]. Further, a 2020 study that used two large US claims databases to analyse the risk of birth defects among 2045 HCQ-exposed and 3198589 unexposed pregnancies observed an increase in the risk of congenital malformations, especially oral clefts, among HCQ-exposed patients. In this study, 54.8 per 1000 infants exposed to HCQ were born with a major congenital malformation compared to 35.3 per 1000 unexposed infants, corresponding to an unadjusted relative risk of 1.51 (95% confidence interval 1.27 to 1.81) and an adjusted relative risk of 1.26 (95% confidence interval 1.04 to 1.54). This increased risk was seen only with HCQ doses of ≥400 mg/day, with the adjusted relative risks being 1.33 (95% confidence interval 1.08 to 1.65) for HCQ doses ≥ 400 mg/day and 0.95 (95% confidence interval 0.60 to 1.50) for HCQ doses <400 mg/day [14]. However, these studies did not take drug concentrations into consideration and analysed a specific ceiling dose, despite variations in the patient and disease factors.

Hydroxychloroquine levels are measured via high-performance liquid chromatography or tandem mass spectrometry in isolation or in combination [15]. Both whole blood and serum levels can be measured, but monitoring whole blood levels is preferred. In a study analysing the utility of HCQ whole blood and serum levels, only whole blood levels were independently associated with SLE activity in the multivariate analysis [16]. Moreover, whole blood measurements may be more reproducible and stable than serum measurements as HCQ diffuses into red blood cells and serum HCQ levels can hence be affected by in vitro factors such as temperature, pH and the duration and force of centrifugation [17,18].

**Figure 1:**
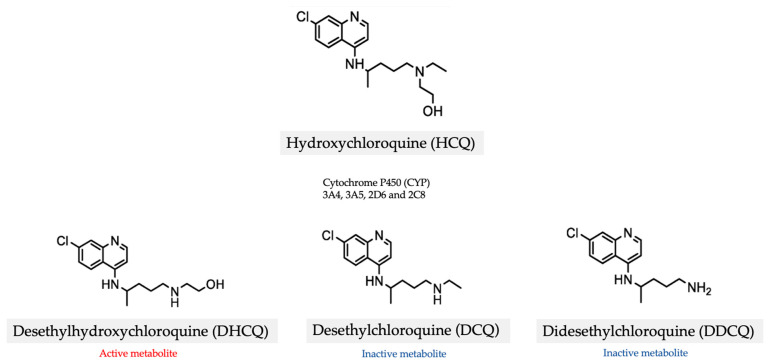
Metabolism of hydroxychloroquine.

HCQ is well absorbed with a high oral bioavailability of 70 to 80% [19]. It has a long and variable plasma elimination half-life due to its large volume of distribution and significant tissue binding [20]. HCQ is mainly metabolized by the cytochrome P450 (*CYP*) *3A4*, *3A5*, *2D6* and *2C8* enzymes into three metabolites in the liver: the active metabolite desethylhydroxychloroquine (DHCQ), as well as the inactive metabolites desethylchloroquine (DCQ), and didesethylchloroquine (DDCQ) [21] (Figure 1). HCQ and these three metabolites are also reversible, competitive *CYP2D6* inhibitors [21].

Due to its unique pharmacokinetic and pharmacogenomic characteristics, blood HCQ concentrations can vary considerably between individuals even at the same dose. A study that analysed 10463 patient samples showed that there was a wide range in the whole blood HCQ levels, from undetectable (<25 ng/mL) to 9502 ng/mL [22].

Although the co-administration of CYP enzyme inhibitors or inducers has been thought to influence HCQ levels, this was not shown in the Plaquenil Lupus Systemic (PLUS) study. Here, there was no significant difference found in the blood HCQ concentrations of 113 patients who received CYP enzyme inhibitors such as erythromycin and antifungal agents, 14 patients who received CYP inducers such as carbamazepine and rifampicin, and 377 patients who received none of these treatments [23]. Similarly, cigarette smoking has been thought to increase the risk and activity of SLE and cutaneous lupus, and reduce the effectiveness of HCQ in cutaneous lupus by inducing CYP enzymes [24]. However, several studies have shown no association between smoking and HCQ concentrations [23,24].

In a study that collected 10523 specimens from 6559 patients receiving HCQ, linear mixed-effect models revealed that the HCQ levels were associated with patient age (93 ± 7 ng/mL per 10 years; *p* < 0.001) and female gender (141 ± 40 ng/mL; *p* < 0.001) [25]. Other factors that influence HCQ levels include *CYP450* polymorphisms and renal function. In a Korean study, 194 patients with SLE were genotyped for four single-nucleotide polymorphisms in CYP2D6*10, CYP3A5*3, and CYP3A4*18B. Their blood HCQ and DHCQ concentrations were measured and their association with the corresponding genotypes was investigated. *CYP2D6* polymorphisms were shown to influence the [DHCQ]:[HCQ] ratio, while the polymorphisms of CYP3A5*3 and CYP3A4*18B did not show any significant association with the [HCQ], [DHCQ], or [DHCQ]:[HCQ] ratio [26]. In a Chinese study, *CYP3A5* polymorphisms predicted a greater risk of skin and mucous membrane adverse drug reactions (ADRs), while *CYP2C8* polymorphisms conferred a greater risk of abnormal renal function and ophthalmic ADRs [27].

The use of HCQ in chronic kidney disease (CKD) is crucial for reducing the risk of further damage to the remaining kidney reserves and delaying progression to end-stage kidney disease [28]. HCQ has been shown to reduce atherosclerosis and vascular stiffness in CKD [29]. Yet, blood HCQ concentrations are profoundly affected by renal function. Several studies including the PLUS study found an inverse correlation between the estimated glomerular filtration rate (eGFR) and blood HCQ concentrations [23,30]. The Joint European League Against Rheumatism and European Renal Association–European Dialysis and Transplant Association (EULAR/ERA–EDTA) guidelines recommend that the maximum dosing of HCQ is 5 mg/kg/day, and that the dose be reduced by 50% when eGFR  < 30 mL/min [31].

However, patients on adjusted HCQ dosing due to renal impairment appear to be underdosed [32]. In a study where patients with renal impairment (serum creatinine 1.4 to 4.9 mg/mL) were dosed with HCQ at 200 mg daily (a 50% reduction from the maximum daily dose of 400 mg), it was found that 60% of them had subtherapeutic HCQ levels of <500 ng/mL. In another Japanese study investigating the pharmacokinetics of HCQ and its metabolites in SLE patients with renal impairment, it was found that renal impairment did not affect the total clearance of HCQ. Hence, the study concluded that dosage reduction is unnecessary in SLE patients with impaired renal function [33]. We emphasise that measuring and monitoring the HCQ levels in patients with CKD is paramount, as HCQ levels predict flares and disease activity [34,35].

However, a consensus on the target therapeutic HCQ level has yet to be established, partly due to a lack of standardisation of HCQ levels across various laboratories. Some studies have suggested 500 to 2000 ng/mL to be the therapeutic range [32,35]. In one such study, stable therapeutic mean HCQ levels >500 ng/mL were associated with no occurrence of disease flares. A systematic review and meta-analysis performed on 17 studies that measured HCQ levels and assessed the SLEDAI scores in patients with SLE found that, among 1223 patients, those with HCQ levels ≥ 750 ng/mL had a 58% lower risk of active disease, and that their SLEDAI scores were an average of 3.2 points lower. Hence, HCQ levels of ≥750 ng/mL were suggested to be a potential therapeutic target [36]. In the Hopkins Lupus Cohort, the therapeutic range was taken to be 1000 to 1500 ng/mL [37]. Most recently, a cross-sectional study found that HCQ levels between 750 and 1100 ng/mL significantly reduced the odds of high lupus disease activity (SLEDAI score ≥ 6) by 76 (odds ratio 0.24, *p* = 0.016) to 90% (odds ratio 0.1, *p* = 0.02) [38].

In the Systemic Lupus International Collaborating Clinics (SLICC) Inception Cohort study, the HCQ levels of patients were measured to identify non-adherence. It showed that severe non-adherence (defined by a serum HCQ level <106 ng/mL for a daily prescribed HCQ dose of 400 mg, and <53 ng/mL for a daily HCQ dose of 200 mg) was associated with an increased risk of SLE flare in the following year, with earlier damage, and with an increased 5-year mortality [39].

Low HCQ blood levels are also associated with thrombotic events. A study of 739 patients with SLE found that lower mean HCQ whole blood levels and lower HCQ whole blood levels closest to the thrombotic event were associated with a higher risk of thrombosis. Mean HCQ whole blood levels of >1068 ng/mL and recent levels of >1192 ng/mL conferred significant protection against thrombosis. Thrombotic events were reduced by 13% for every 200 ng/mL increase in the mean HCQ blood level, and were reduced by 69% in patients with higher mean HCQ blood levels >1068 ng/mL, versus <648 ng/mL (rate ratio 0.31; *p =* 0.024) [40].

HCQ levels can also be used to predict retinal toxicity, especially in patients with a long duration of treatment, a high cumulative dose, CKD, concomitant tamoxifen use, or pre-existing macular disease. In a study evaluating 537 SLE patients from the Hopkins Lupus Cohort, it was found that both the mean and maximum HCQ blood levels predicted later HCQ retinopathy. Patients had a higher risk of retinopathy with higher maximum HCQ blood levels (1.2% in 0 to 1182 ng/mL, 4.8% in 1183 to 1752 ng/mL, 6.7% in 1753 to 6281 ng/mL) and higher mean HCQ blood levels (1.2% in 0 to 741 ng/mL, 3.7% in 741.5 to 1176.5 ng/mL, 7.9% in 1177 to 3513 ng/mL) [41]. However, HCQ whole blood levels did not correlate with the QTc intervals regardless of the dose of HCQ prescribed in a prospective study of 84 SLE patients [42].

Physiologic changes during pregnancy, such as an increase in the volume of distribution, may also substantially alter the drug pharmacokinetics of HCQ and hence its drug levels. An observational study of pregnant patients with rheumatic diseases taking HCQ showed that there was significant variability in the HCQ concentrations at each trimester. Both high (>500 ng/mL) and low (<100 ng/mL) mean HCQ levels were associated with preterm birth and disease activity, and the optimal HCQ level appeared to be in the low–moderate range (101 to 500 ng/mL) in that cohort [43]. This suggests that the relationship between HCQ drug levels and neonatal outcomes are complex and not linear, and that further studies need to be performed to clarify the role of monitoring HCQ levels in lupus pregnancies.

## 4. Mycophenolate

Mycophenolate is a potent inhibitor of lymphocyte proliferation. It reversibly inhibits inosine monophosphate dehydrogenase (IMPDH), which catalyses the conversion of IMP to guanosine monophosphate, leading to decreased B- and T-lymphocyte proliferation and decreased antibody production [44].

Mycophenolate is available in two formulations: mycophenolate mofetil (MMF) and enteric-coated mycophenolate sodium (EC-MPS). Both are hydrolysed into mycophenolic acid (MPA), which is the pharmacologically active metabolite. Both formulations are similar in structure, with the same active moiety but different side chains. MPS is enteric coated and is hence protected against the stomach acids, causing delayed release into the small intestine [45]. Hence, the time to maximum concentration is longer for EC-MPS (varies between 0 and 6 h) compared to that of MMF (varies between 0.5 and 2 h) [46]. Due to its delayed release, adverse gastrointestinal effects, especially diarrhoea, are reduced with the use of EC-MPS. However, EC-MPS has higher pharmacokinetic variability compared with MMF, which is reflected in an unpredictable and irregular concentration–time profile and a higher variability in the main pharmacokinetic parameters [47]. TDM for EC-MPS is not clinically feasible at present.

More than 90% of MPA is metabolized via glucuronidation by uridine 5′-diphospho-glucuronosyltransferases (UGTs) into the inactive metabolite 7-O-glucuronide (MPAG); this mainly occurs in the liver, but can also occur in the intestines and kidneys [48]. Thereafter, MPAG is hydrolysed back to MPA via enterohepatic recirculation, resulting in a 2nd peak in the MPA concentration. Approximately 97% of MPA is bound to albumin, so the levels of free MPA may be increased in patients with hepatic dysfunction or hypoalbuminemia.

In a study exploring the relationship between pharmacogenomic variations and MPA exposure in patients with lupus nephritis on MMF, it was found that the rs2273697 genotype adenine–guanine (A/G) in the *ABCC2* gene was associated with significantly lower MPA drug exposure when compared with genotype guanine–guanine (G/G). The same study showed no association between UGT polymorphisms and MPA exposure [49].

Due to large inter-individual variability in the pharmacokinetics of MPA in different patients, the fixed dosing of MPA can lead to highly variable drug exposure. Studies have demonstrated that in transplant patients, the MPA exposure can vary 10-fold amongst patients treated with a daily dose of 2 g of MMF [50]. Factors affecting this large inter-individual variability include ethnicity and other pharmacogenetic factors, renal function, liver function, serum albumin levels and the use of concomitant drugs such as proton pump inhibitors and iron oxide [51]. Asian renal transplant patients have been reported to have higher MPA exposure compared with their white counterparts, and the optimal dose of MMF has been shown to be 20 to 46% lower in Asian transplant patients than in white patients [52]. Renal impairment has also been shown to lead to higher exposure to MPA due to the reduced elimination of MPA [53]. Given that multiple factors can affect the pharmacokinetic profiles of MPA, resulting in high inter-individual variability in MPA exposure and variation in treatment responses, TDM of MPA should be performed to maximise efficacy and minimise toxicities.

Data on TDM of MPA comes mainly from the literature on transplants, but results can also be applied to MPA use in patients with lupus nephritis. In a study assessing whether pharmacokinetic monitoring helps to optimize the dosing of MPA in an Asian population with lupus nephritis, the mean area under the concentration–time curve (AUC) for the MPA of responders was found to be significantly higher than those of non-responders [54]. A cross-sectional study of children with SLE also showed that the mean MPA 12 h area under concentration–time curve (AUC_0–12_) concentrations were significantly lower in patients with active disease (SLEDAI score > 6) than those with inactive disease (SLEDAI score < 6) [55].

The AUC_0–12_ is considered the criterion standard for the monitoring of MPA and is a good reflection of exposure to the drug over the entire dosing period. However, AUC calculation requires multiple blood samplings, which may not be feasible or practical in real-life clinical practice [56]. Hence, limited sampling strategies (LSSs) that estimate AUC_0–12_ from a small number of samples have been proposed. LSSs are developed using multiple regression analysis and Bayesian analysis, and are recommended for use only in populations for which the model has been developed [57].

Unfortunately, MPA trough levels weakly correlate with MPA-AUC in renal transplant patients [58,59]. The few studies in patients with lupus nephritis that showed a positive correlation were limited by their small sample sizes [54,60]. A study in patients with lupus nephritis that used regression analysis to examine the relationship between the MPA trough level and AUC also showed no strong correlation [61]. The predictive value of the MPA trough level, versus MPA-AUC, needs to be studied in greater detail in future studies.

In renal transplant patients, an AUC_0–12_ of 30 to 60 mg × h/L has been directly correlated with the minimisation of renal allograft rejection [62]. In lupus nephritis, different threshold targets have been proposed. A Thai study suggested maintaining AUC levels above 45 mg × h/L [54], while another Australian study showed good renal responses at 1 year in patients with AUC ≥ 30 mg × h/L [63]. Another study from the Netherlands showed that a target AUC_0–12_ of 60 to 90 mg × h/L resulted in good renal outcomes, with 18.7% of patients achieving partial remission and 68.8% achieving complete remission at 12 months of follow-up [64]. An older review from 2015 that compared the results from previous TDM studies suggested target AUC threshold values of 30 to 45 mg × h/L and a target trough threshold of 3 mg/L [65].

## 5. Calcineurin Inhibitors

Cyclosporine and tacrolimus selectively inhibit calcineurin and have similar suppressive effects on cell-mediated and humoral immune responses. They have been the mainstays of immunosuppression in solid organ transplantation, but are also used in the treatment of SLE. A large trial in Chinese patients with lupus nephritis has shown a higher overall response rate in patients with a multitarget regimen of tacrolimus in combination with MMF compared with cyclophosphamide [66]. As an extension of this trial, it was also shown that patients continued on this multitarget regimen had similar rates of relapse to those who were given cyclophosphamide for induction and received azathioprine for maintenance [67].

Both cyclosporine and tacrolimus bind with high affinity to cytoplasmic receptors, namely the cyclophilins and FK-binding proteins, respectively. The resultant drug–receptor complex then competitively binds to and inhibits calcineurin [68]. Calcineurin is a calcium- and calmodulin-dependent phosphatase that removes phosphate from a family of transcription factors (NF-AT) which then translocate into the nucleus and cause the transcription of cytokines such as interleukin (IL)-2, tumour necrosis factor (TNF)-alpha, IL-3, IL-4, granulocyte-macrophage colony-stimulating factor, and interferon-gamma. Hence, inhibiting calcineurin ultimately results in reduced T cell activation, proliferation and differentiation [69]. In addition to its immunomodulatory effects, calcineurin inhibitors are also able to decrease proteinuria via direct podocyte stabilisation and afferent arteriole vasoconstriction.

Both drugs reach peak blood concentrations after 1 to 8 h. They are lipophilic and undergo extensive distribution. Cyclosporine binds mainly to lipoproteins, while approximately 99% of tacrolimus binds to proteins in the plasma [68]. Both drugs are extensively metabolised by *CYP3A* enzymes in the liver. P-glycoprotein prevents drug absorption from the gut by promoting efflux into the lumen of the intestine. They are then eliminated through excretion in the bile.

The side effects of cyclosporine and tacrolimus are similar. Firstly, they can cause nephrotoxicity in the form of an acute rise in the plasma creatinine levels. This is largely reversible via dose reduction but can occasionally progress to CKD, which is usually irreversible [68,69]. Both drugs can also cause hypertension via renal vasoconstriction and sodium retention. In addition, they can also cause neurotoxicity including tremors, and encephalopathy presenting as headache and seizures [69]. Calcineurin inhibitors have also been associated with metabolic effects such as diabetes mellitus, hyperlipidaemia, gout and other side effects such as hirsutism and gum hypertrophy.

Blood concentrations of tacrolimus are strongly influenced by *CYP3A5* enzymes. In transplant patients, more than 50 studies have found that *CYP3A5* expressors who are extensive and intermediate metabolisers (i.e., patients carrying the *1 allele e.g., CYP3A5*1/*1 or CYP3A5*1/*3 genotype) have significantly lower dose-adjusted trough concentrations of tacrolimus compared to *CYP3A5* non-expressors who are poor metabolisers (e.g., CYP3A5*3/*3 genotype) [70]. The Clinical Pharmacogenetics Implementation Consortium (CPIC) guidelines have recommended a higher starting dose of 1.5 to 2 times the standard dose for patients who are extensive and intermediate metabolisers. There are also multiple drug–drug interactions between calcineurin inhibitors and other medications. *CYP3A4* inhibitors such as colchicine and oral contraceptive pills can increase their plasma concentrations, while *CYP3A4* inducers such as anti-seizure medications and rifampicin can decrease their plasma concentrations.

Data on the therapeutic drug monitoring of calcineurin inhibitors in SLE are sparse. A Montreal study was performed on patients from the Lupus prEGnAnCY (LEGACY) cohort, a prospective cohort enrolling SLE pregnancies at Systemic Lupus International Collaborating Clinics. The preliminary results showed that in pregnancies with sub-therapeutic tacrolimus levels, 50% were not in the Lupus Low Disease Activity State (LLDAS), while all pregnancies with therapeutic or supratherapeutic tacrolimus levels were in the LLDAS [71].

Cyclosporine can be measured using the residual or trough concentration at 12 h (C_0_), the peak concentration 2 h post dose (C_2_), or the AUC. It has been shown that C_0_ correlates poorly with nephrotoxicity and acute rejection in renal transplant patients [72]. Both nephrotoxicity and acute rejection are better correlated with the AUC between 0 to 4 h or 0 to 12 h (AUC_0–4_, AUC_0–12_) [73], but this again requires the collection of multiple blood samples, which is cumbersome in routine clinical practice. To date, C_2_ appears to be the best single-time-point predictor of the AUC, and has shown to be highly predictive of acute rejection in de novo transplant recipients [74,75].

Likewise for tacrolimus, the best marker of drug exposure is the AUC [76]. However, unlike cyclosporine, the correlation between C_2_ and the AUC is not superior to that between C_0_ and the AUC [77]. Hence, measuring the tacrolimus trough level is the preferred choice of TDM for tacrolimus.

For transplant patients, the target cyclosporine and tacrolimus trough concentrations are dependent on the type of transplant and the time post transplant. In the multitarget lupus nephritis trial that used MMF and tacrolimus 4 g/day, the tacrolimus trough levels were kept between 5 and 7 ng/mL, with participants reaching an average of 5.24 to 5.5 ng/mL over weeks 4 to 24 [66]. There are limited data on the target cyclosporine C_0_ or C_2_ levels in patients with lupus nephritis.

Voclosporin has also been shown to be an effective agent for the treatment of lupus nephritis [78,79]. There is no need for TDM when using voclosporin as it has a predictable pharmacokinetic profile. A population pharmacokinetic analysis in patients with lupus nephritis showed that voclosporin has a linear pharmacokinetic profile. Factors such as sex, body weight, age, serum albumin, total bilirubin and eGFR did not have any significant or clinically relevant effect on its pharmacokinetic parameters. This enables a pharmacodynamic rather than pharmacokinetic approach to dosing, with the dose adjusted in response to decreases in the eGFR [80].

## 6. Azathioprine (AZA)

**Figure 2:**
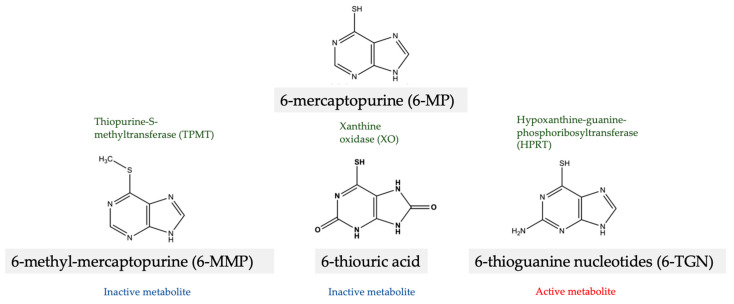
Metabolism of 6-mercaptopurine (6-MP).

Azathioprine is a prodrug that is rapidly converted to 6-mercaptopurine (6-MP) by a non-enzymatic process. 6-MP is then metabolized in the liver and gut via three major pathways: (1) by xanthine oxidase to inactive 6-thiouric acid, (2) by thiopurine-S-methyltransferase (TPMT) to inactive 6-methyl-mercaptopurine (6-MMP) and (3) by hypoxanthine-guanine-phosphoribosyltransferase (HPRT) to active 6-thioguanine nucleotides (6-TGN) (Figure 2).

The principal mechanism for the immunosuppressive effects of AZA is thought to be via the incorporation of 6-TGN into nucleic acids and the inhibition of purine and protein synthesis in lymphocytes [81]. AZA and 6-MP also inhibit the proliferation of T and B lymphocytes, leading to the decreased production of cytotoxic T lymphocytes and plasma cells. High levels of 6-MMP can cause hepatoxicity while high levels of 6-TGN can lead to bone marrow suppression and cytopenia [82].

TPMT activity can be evaluated through either the genotyping of the TPMT gene or by directly measuring the TPMT activity in red blood cells in vitro [83]. Polymorphisms in the TPMT gene can result in differing levels of TPMT enzyme activity. Low or absent TPMT enzyme activity can result in the overproduction of 6-TGN, leading to bone marrow suppression, while high TPMT enzyme activity can result in greater 6-MMP production at the expense of 6-TGN, leading to reduced efficacy [84]. The TPMT gene is highly polymorphic, with over 40 reported variant alleles [85]. The TPMT*1 is a wild-type allele in which no variants are detected and that is associated with normal enzyme activity. Most individuals have normal enzyme activity and are called TPMT normal metabolisers. Approximately 4% to 11% of the population are heterozygous for a variant TPMT allele and have intermediate enzyme activity (TPMT intermediate metabolisers), while around 0.3% are homozygous for variant TMPT alleles and have very low or absent enzyme activity (TPMT poor metabolisers) [86].

In the absence of TPMT variants, myelosuppression is still observed clinically. This can be attributed to diphosphate-linked moiety X motif 15 (NUDT15), which converts the active 6-TGN to inactive metabolites. Similarly, in individuals who have reduced or absent NUDT15 activity (i.e., intermediate or poor metabolisers, respectively), there is an overproduction of 6-TGN, resulting in a higher risk of myelosuppression.

While TPMT deficiency is the primary genetic cause of thiopurine intolerance in European and African people, NUDT15 deficiency is common in Asian and Hispanic people [83]. While there is scant data for SLE, randomized controlled trials in IBD patients have shown that there is a reduction in azathioprine-induced myelosuppression with genotype-guided prescribing compared to the standard of care [86,87]. The Clinical Pharmacogenetics Implementation Consortium (CPIC) guidelines have recommended dosing adjustments based on individuals’ TPMT and NUDT15 metaboliser status [88].

Furthermore, the TDM of thiopurine metabolites (i.e., 6-TGN and 6-MMP) can provide further information on the adjustment of the azathioprine dose. Several studies have suggested that higher levels of 6-TGN are associated with both the clinical response and myelosuppression. A meta-analysis evaluating the relationship between 6-TGN levels and clinical remission in IBD patients found that 6-TGN levels of 235 to 450 pmol/8 × 10^8^ RBCs have been correlated with the clinical efficacy of thiopurines [89]. These patients were four times likely to be in clinical remission. In dermatology, 150 to 300 pmol/8 × 10^8^ RBCs have been suggested to be the optimal levels for disease remission in immunobullous diseases [90]. On the other hand, elevated levels of 6-MMP > 5700 pmol/8 × 10^8^ RBCs are associated with hepatoxicity [91].

For SLE, the optimal levels of 6-TGN associated with the clinical response have not yet been established. Preliminary results from the LEGACY cohort showed that only 43% of pregnancies with undetectable or barely detectable 6-TG levels were in LLDAS, compared to 71% of those with higher 6-TG levels [71]. In a study evaluating the utility of drug escalation based on the metabolite levels of SLE patients, 21 out of 50 patients achieved clinical responses; 13 of these patients had 6-TGN levels less than 235 pmol/8 × 10^8^ RBCs. Yet, 10 patients who did not have a clinical response at 6 months had either achieved therapeutic IBD 6-TGN levels of more than 235 pmol/8 × 10^8^ RBCs or received a maximum AZA dose ≥ 3.5 mg/kg [92]. This shows that clinical responses in SLE can occur at levels of 6-TGN lower than the target range established for IBD, and that more work needs to be done to establish the optimal metabolite levels for SLE patients.

In a retrospective study of SLE patients, half of all patients on AZA ceased treatment due to presumed inefficacy or toxicity [93]. This study’s authors proposed that measuring AZA metabolites would enable true treatment refractoriness to be distinguished from the under-dosing of AZA, and drug toxicity to be distinguished from lupus-related leukopenia in these patients. To achieve this, prospective studies looking into establishing well-defined therapeutic metabolite targets in SLE and strategy trials adopting metabolite-based dosing algorithms are required. This can then enable clinicians to optimise theAZA dose and prevent premature treatment escalation.

## 7. Summary

TDM is especially important when there are complicated pharmacokinetics and/or patient-related factors that influence the therapeutic or toxic effects of the drug (Table 1). By taking these factors into account, TDM can empower physicians to personalise therapy by prescribing appropriate doses in SLE patients to achieve therapeutic efficacy yet minimise side effects at the same time. While biologic therapies are increasingly used in the SLE treatment armamentarium, oral immunosuppressive and immunomodulatory therapies, especially HCQ, remain the mainstay in the majority of patients at present. However, resources need to be expended to maintain a TDM service, including administrative efforts to maintain an organised and well-oiled system of appropriately timed blood sample drug collection, manpower for the supervision and review of drug levels, as well as the monitoring of patients’ responses to treatment. An analysis of the cost effectiveness of such a service would be relevant to its sustainability and scalability. Unfortunately, data in SLE are lacking and trailing behind the literature on transplants and IBD. More studies are required to enable clinicians to individualise these therapies via TDM in SLE.

## Figures and Tables

**Table 1:** Summary of medications for systemic lupus erythematosus (SLE).

Drug	Hydroxychloroquine	Mycophenolate	Calcineurin Inhibitors	Azathioprine
Indication in SLE	- Anchor drug in SLE - Reduces lupus flares [6] and damage accrual - Prevents thrombosis [9] - Improves metabolic profile [94] - Reduces rates of preterm births, congenital heart block and pre-eclampsia [95]	- Induction and maintenance therapy in lupus nephritis [96,97] - Reduces glucocorticoid requirements and flares in extra-renal manifestations [98,99]	Induction and maintenance therapy in lupus nephritis as part of multitarget regimen [66,67]	Maintenance therapy for SLE
Key metabolisers	*CYP3A4*, *3A5*, *2D6*, *2C8* [21]	UDP-glucuronosyltransferases [47]	*CYP3A4*, *3A5*	Xanthine oxidase, TPMT, HPRT
Factors affecting blood concentration (besides dose and adherence)	- Age [25] - Gender [25] - *CYP450* polymorphisms [26,27] - Renal function [23,30]	- Ethnicity [52] - Renal function [53] - Liver function [51] - Serum albumin levels [51] - Concomitant drugs (e.g., proton pump inhibitors, iron oxide) [51]	- *CYP3A* inhibitors or inducers including dietary polyphenols - *CYP3A5* polymorphisms [70]	- Xanthine oxidase inhibitor - TPMT/NUDT15 polymorphisms [84]
Utility of monitoring	- Identify non-adherence, which predicts damage [39] and mortality - Predicts lupus flares and treatment failure [34,35] - Predicts thrombotic events [40] - Predicts HCQ retinopathy [41]	Determine appropriate dose to maximise efficacy and minimise toxicities	Determine appropriate dose to maximise efficacy and minimise toxicities	- 6-TGN levels can predict clinical response [89,90] - 6-MMP levels can predict hepatotoxicity [91]
Drug levels of clinical importance	- HCQ levels ≥ 750 ng/mL associated with 58% lower risk of active disease [36] - HCQ levels 750 to 1100 ng/mL associated with reduced odds of high lupus disease activity by 76 to 90% [38] - Mean HCQ levels > 1068 ng/mL, versus <648 ng/mL, reduce thrombotic events by 69% [40] - Mean HCQ levels > 1177 ng/mL associated with doubling of risk of retinopathy compared to 742 to 1176 ng/mL (7.9% versus 3.7%) [41]	- AUC_0–12_ of 30 to 60 mg × h/l associated with 68.8% complete renal remission of lupus nephritis at 12 months [64] - AUC threshold values of 30 to 45 mg × h/L and target trough threshold of 3 mg/L suggested by some authors [65]	- Tacrolimus trough levels of 5 to 7 ng/mL were maintained in the multitarget lupus nephritis trial [66] - Cyclosporine: no data	- 6-TGN levels of 235 to 450 pmol/8 × 10^8^ RBCs associated with clinical remission in IBD patients [89] - 6-MMP levels > 5700 pmol/8 × 10^8^ RBCs associated with hepatoxicity [91] - Optimal levels not yet established in SLE patients
Unknown gaps	- Pharmacokinetics, pharmacodynamics and optimal exposure of HCQ during pregnancy - Utility in predicting other adverse events besides retinopathy	Correlation between MPA trough level and MPA-AUC	Association of TDM with SLE outcomes	Target 6-TGN levels to predict therapeutic efficacy and toxicities in SLE patients

## Data Availability

Not application.
